# Factors Associated with Correct and Consistent Insecticide Treated Curtain Use in Iquitos, Peru

**DOI:** 10.1371/journal.pntd.0004409

**Published:** 2016-03-11

**Authors:** Valerie A. Paz-Soldan, Karin Bauer, Amy C. Morrison, Jhonny J. Cordova Lopez, Kiyohiko Izumi, Thomas W. Scott, John P. Elder, Neal Alexander, Eric S. Halsey, Philip J. McCall, Audrey Lenhart

**Affiliations:** 1 Department of Global Health Systems and Development, Tulane University School of Public Health and Tropical Medicine, New Orleans, Louisiana, United States of America; 2 United States Naval Medical Research Unit No. 6 (NAMRU-6), Iquitos Laboratory, Iquitos, Peru; 3 Department of Entomology and Nematology, University of California Davis, Davis, California, United States of America; 4 Division of Health Promotion and Behavioral Sciences, Graduate School of Public Health, San Diego State University, San Diego, California, United States of America; 5 MRC Tropical Epidemiology Group and Department of Infectious Disease Epidemiology, London School of Hygiene and Tropical Medicine, London, United Kingdom; 6 Malaria Branch, United States Centers for Disease Control and Prevention, Atlanta, Georgia, United States of America; 7 Department of Vector Biology, London School of Hygiene and Tropical Medicine, London, United Kingdom; 8 Entomology Branch, Division of Parasitic Diseases and Malaria, United States Centers for Disease Control and Prevention, Atlanta, Georgia, United States of America; Mahidol University, THAILAND

## Abstract

Dengue is an arthropod-borne virus of great public health importance, and control of its mosquito vectors is currently the only available method for prevention. Previous research has suggested that insecticide treated curtains (ITCs) can lower dengue vector infestations in houses. This observational study investigated individual and household-level socio-demographic factors associated with correct and consistent use of ITCs in Iquitos, Peru. A baseline knowledge, attitudes, and practices (KAP) survey was administered to 1,333 study participants, and ITCs were then distributed to 593 households as part of a cluster-randomized trial. Follow up KAP surveys and ITC-monitoring checklists were conducted at 9, 18, and 27 months post-ITC distribution. At 9 months post-distribution, almost 70% of ITCs were hanging properly (e.g. hanging fully extended or tied up), particularly those hung on walls compared to other locations. Proper ITC hanging dropped at 18 months to 45.7%. The odds of hanging ITCs correctly and consistently were significantly greater among those participants who were housewives, knew three or more correct symptoms of dengue and at least one correct treatment for dengue, knew a relative or close friend who had had dengue, had children sleeping under a mosquito net, or perceived a change in the amount of mosquitoes in the home. Additionally, the odds of recommending ITCs in the future were significantly greater among those who perceived a change in the amount of mosquitoes in the home (e.g. perceived the ITCs to be effective). Despite various challenges associated with the sustained effectiveness of the selected ITCs, almost half of the ITCs were still hanging at 18 months, suggesting a feasible vector control strategy for sustained community use.

## Author Summary

Dengue is an arthropod-borne virus of great public health importance. Vector control is currently the only available method for dengue prevention. This cluster-randomized trial investigated individual and household-level socio-demographic factors associated with correct and consistent use of insecticide-treated curtains (ITCs)—one promising vector control method—in Iquitos, Peru. Most people preferred to hang the ITCs in doorways and as room dividers, but also hung them as curtains on windows and on their walls. We assessed who still had their ITCs hanging or tied up at 9 months and 18 months after distribution, and found that use of the ITCs decreased over time to about half. When we explored who was more likely to be using the ITCs correctly (having them hanging in place, or tied up in place, or washed without bleach and avoiding direct sunlight), we found that those who knew more about dengue, knew someone who had dengue, had young children in their homes sleeping under an insecticide treated mosquito net, or who perceived the ITCs to work well, were more likely to be using their ITCs than others. Despite various challenges in sustained ITC effectiveness in this study, the fact that almost half of the homes still had the ITCs hanging at 18 months suggests this vector control strategy is feasible for long term community use.

## Introduction

Dengue viruses, transmitted primarily by the diurnal-biting mosquito, *Aedes aegypti*, are the cause of more human morbidity and mortality than any other arthropod-borne virus, with an estimated 96 million apparent infections and an additional 294 million inapparent infections occurring globally in 2010 [1]. The Americas are one of the most affected areas in the world, and in 2013 had the largest number of cases ever reported in that region—2.3 million—of which 37,692 were severe and 1,208 resulted in death [2] Peru bears a substantial portion of the disease burden in the Americas, with 11,816 cases and 16 deaths reported in 2013 [3]. In particular, the Department of Loreto, where this study was conducted, experienced nearly a third of the country’s cases in 2013, with 3,542 cases reported [3].

While significant advances are underway in dengue vaccine development, vector control remains the only current option for prevention of dengue infection [4,5], and it is likely to remain necessary once vaccines become available both in the context of integrated dengue control programs and to prevent other *Aedes aegypti* transmitted infections such as the rapidly increasing chikungunya virus. Vector control strategies most commonly target the immature stages of the mosquito, as *Ae*. *aegypti* preferentially breed in containers in close proximity to human habitations. Container control interventions at most reduce vector density and do not impact adult mosquito longevity, which limits their potential to reduce dengue transmission. Interventions targeting the adult stage of the mosquito are often only used in outbreak situations due to their limited efficacy and residuality, as well as their comparatively labor intensive requirements [6–9]. For this reason, long-lasting interventions targeting the adult stage of the mosquito are attractive, and the use of insecticide-treated curtains (ITCs) offers one promising household-level strategy to achieve this. The use of ITCs combined with insecticide-treated container covers has been demonstrated to decrease significantly both the Breteau Index (BI) (number of positive containers/100 households) and pupae per person index (PPI), with evidence that a protective spillover effect from the intervention occurs in neighboring untreated areas [10–15].

The effectiveness of any intervention relies on more than just its availability. Successful implementation requires full acceptance by key community leaders and members, as well as correct and consistent use of the intervention [16–18]. Numerous studies have been conducted about the use of insecticide-treated materials (ITMs) as vector control tools for malaria prevention [19–27], but the use ITCs for dengue prevention has been evaluated in only a few contexts. Those studies reported high acceptability of ITCs [13,15], particularly among those families who had previous experience with dengue infection [13] and who perceived the ITCs to be effective [28]. Continued ITC use was reported to be higher among families who had been resident in their home for more than five years and families that had previously used decorative curtains in their homes [28].

An attractive aspect of using ITCs to suppress dengue vector populations is that they comprise a largely passive intervention: the ITCs simply hang inside a house and beyond acceptance of their presence they require minimal behavior change on the part of the householders. Understanding the socio-demographic factors and knowledge, attitudes and practices associated with correct and consistent ITC use can shed light on how to maximize the effectiveness of future ITC intervention programs. A systematic review of the effectiveness of 21 dengue fever prevention programs from around the world revealed that an understanding of the link between human behavior and correct and effective vector control programs was missing [16]. Additionally, a more recent study focusing on the individual determinants of insecticide-treated bednets use—a similar vector control strategy—identified a gap in understanding which behavioral mechanisms and educational tools were associated with successful ITC program implementation [27]. This study aims to fill these gaps with respect to the effectiveness of ITC interventions for dengue control.

As part of a cluster-randomized trial examining whether ITCs could reduce dengue transmission, the objective of this study was to determine the individual and household-level socio-demographic factors that are associated with the correct and consistent use of ITCs in Iquitos, Peru. Correct and consistent use was defined for this study as 1) ITCs *observed* as hanging properly—loosely and extended—at the time of visit, 2) ITCs *observed* to be tied up, but still in their place, at the time of visit, and 3) ITCs *reported* as washed correctly–with only water and/or mild soap and hung to dry in the shade.

## Methods

### Study Setting

This observational study was conducted in an urban part of the San Juan district population ~102,000) of Iquitos, Peru (population ~430,000)—a large, geographically isolated city in the middle of the Amazon rainforest, accessible only by boat or plane [29]. Dengue epidemiology has been extensively studied in Iquitos since 1999 by the University of California at Davis/U.S. Naval Medical Research Unit 6-Iquitos group [30–39].

### Study Design

This study is part of a larger cluster-randomized controlled trial, initiated in October 2009, to measure whether ITCs can reduce dengue transmission and dengue vector activity in 10 treatment clusters compared to 10 control clusters of approximately 70 households each (2–3 city blocks). ITCs were an official WHO-recognized brand of long-lasting insecticide (deltamethrin) treated material.

Based on formative research conducted prior to the study, we estimated that each household would request approximately 5 ITCs, but participants could request as many as they wanted. There were three colors to choose from–pink, sky blue and dark blue–and the ITCs were lacey, since there was consensus at the formative stage that this is what individuals preferred. Study staff hung the ITCs in locations suggested by householders, which were documented (i.e., doorways, walls, windows, as room dividers). A member of the study team distributed an information sheet about the ITCs and discussed their correct use (i.e., hanging loosely) and upkeep (i.e., not washed with bleach, kept out of direct sunlight when being dried, mend holes if holes form) with the householders. Due to an unexpected problem with ITC losing bioefficacy after 3–6 months, the research team removed all ITCs from houses 12 months after distribution and re-treated them with deltamethrin, and then returned them to their locations in the houses.

One month prior to ITC distribution, a baseline survey was administered to 1,333 individuals in intervention and control households, assessing knowledge, attitudes, and practices (KAP) associated with dengue and mosquito control [40]. A total of 3,178 ITCs were then distributed to the 593 intervention households between November and December 2009, with an additional 1,049 ITCs distributed to the intervention homes that requested more 9 months later.

Follow up KAP surveys were conducted at 9 and 27 months post-ITC distribution with the same household respondent, when questions regarding barriers and motivators to regular ITC use were included (although there were no KAP related interventions, we expected possible knowledge improvement from the regular presence of our research team in the community monitoring ITCs and answering questions). ITC-monitoring checklists were also conducted at 9 and 18 months post-ITC distribution, when “correct and consistent use” of the ITCs, as has been defined for this study, was verified by direct observation by a 7-membered research team. If individuals wanted more ITCs for their homes at the 18 months, more ITCs were distributed at that time. Additionally, focus group discussions with a small sub-sample were conducted at 6 and 12 months to understand more about the participants’ experience using the ITCs–findings from the focus groups are being published separately. For the KAP surveys and ITC-monitoring checklists, we sought to interview the same individual at the home who was responsible for the ITCs–which was determined by asking who makes the decisions in the home (most commonly the person who managed all aspects of the household, e.g. housewife). If the person identified as responsible for the ITCs was not at home during follow-up visits, additional visits were made in the morning, afternoon, and evening hours a minimum of 3 times and a maximum of 8 times. If repeated visits were unsuccessful in locating the person responsible, then the home was removed from the analysis. All tables report whether data came at 9, 18 or 27 months, unless it was socio-demographic and dengue-related knowledge or preventive practices which all come from the baseline KAP.

### Monitoring of ITC Use

The hanging, condition and upkeep status of the ITCs was observed at 9 and 18 months after distribution using a monitoring checklist based on direct observation. To assess hanging status, our trained research team members directly observed, for each ITC, whether it was: 1) hanging correctly, 2) hanging in place but tied up, 3) being washed, 4) being stored, 5) other location, or 6) missing (see Table 1 for a full list of variables and their definitions). The condition of the ITC was also directly observed by our team, and recorded as excellent, good, poor, or missing. To ensure consistency in the responses recorded by our research team members, during training the team practiced recording their observations about ITCs in different conditions until their reports were consistent with each other. To assess the upkeep status, the householder was asked how the ITC had been washed, and who had washed it, as well as whether it had required mending–and if so, it was checked for having been mended by our research team. Other relevant variables and their definitions are also included in Table 1.

**Table 1:** Variable list: Definition of variables used in the analyses.

General category and variable name		Definition
**Hanging status (observed)**		
	Hanging correctly	50% or more of the ITCs in the house were hanging fully extended—either above or touching the ground.–at the time of our observation visit
	Tied up	50% or more of the ITCs in the house were hanging in place but tied up when our team came to observe, and reported to be tied up any amount of time throughout a day. The median number of hours these were reported tied up was 12 hours, with the range being 1 to 24 hours. The main reason for being tied up was that in some locations there was heavy transit or it was inconvenient to keep them fully extended all day (e.g., in a doorway).
	Being washed	Observed to be in the wash or hanging to dry.
	Stored	Observed to be present in the house, but not hanging nor in the laundry (e.g., stored in a cabinet or closet).
	Other location	Being used in a different way than originally placed (e.g., as a mosquito net, closet door, tablecloth).
	Missing	Missing from the house.
**Condition status (observed)**		
	Excellent	Looks new, not dirty, and not torn.
	Good	Well conserved, clean, and any tears had been mended. Clearly used, but in good shape.
	Poor	Dirty, ripped, and broken. Clearly used, but no apparent effort to keep the ITC in good condition.
	Missing ITC	Missing from the house.
**Upkeep status (self-reported)**		
	Washed correctly	Washed only in water and/or with mild soaps and dried in the shade.
	Washed incorrectly	Dried in the sun or washed with bleach or detergent.
	Not washed	Not washed at all.
	Mended	ITC got a tear or rip somewhere which respondent repaired. This was verified by *observation* by our team.
	Missing ITC	Missing from the house.
**Knowledge of dengue**		
	Knows three or more correct symptoms of dengue	Respondents were able to name at least 3 typical symptoms in dengue patients (i.e., fever, headache, body ache, chills, nausea, vomiting, joint pain, or hemorrhage).
	Knows at least one correct treatment	Respondents knew of at least one correct treatment for dengue symptom relief (i.e., take acetaminophen, visit a health center, or drink plenty of liquids).
	Knows three or more correct protection methods	Respondents were able to name at least 3 appropriate household practices that could prevent dengue (prevent mosquito bites, use products against mosquitoes at home, use repellent, clean the house, spray/fumigate the house, dispose of useless containers, or put lids on water containers).
**Perception of risk for dengue**		
	Knows a relative or close friend who has had dengue	Respondents know a relative or close friend who has had dengue in the past.
**Mosquito bite prevention practices**		
	Children sleep under a mosquito net	Respondents’ children, of any age, sleep under a mosquito net.
**Perceived effectiveness**		
	Saw a change in the amount of mosquitoes in the home	Respondents were asked if there was a change in the amount of mosquitoes in the home since the ITCs were hung. The responses were collapsed into “yes/only for a few months” vs. “no”.
	Would recommend ITCs	Respondents were asked if they would recommend the ITCs to family or friends.

### Data Analysis

STATA 11.0 was used to estimate the means (for continuous variables) and frequencies (if categorical) of social, economic, and demographic variables, as well as the main variables of interest (hanging, condition and upkeep status). Principal component analysis (PCA) was used to create the socioeconomic status (SES) variable, where 50% above the mean was defined as higher SES and less than or equal to 50% was defined as lower SES. Components of the PCA included: exterior wall material, interior wall material, roof material, floor material, window material, cooking material, has fixed telephone line, has electricity, and number of refrigerators, televisions, DVD, computer, radio, washing machine, cars, rooms in the home. Because each home had more than one ITC, the dependent variables of interest—ITCs hanging correctly, tying ITCs up, and washing ITCs correctly—were made dichotomous by classifying those homes with 50 percent or more ITCs hanging, tied up or washed correctly as ‘correct’, whereas those with 49 percent or less were classified as ‘not correct’.

Chi-square tests were conducted to determine whether differences observed in the categorical variables of knowledge, perception of risk, prevention practices, and perceived effectiveness were statistically different between those households: 1) that had ITCs hanging fully extended vs. tied up or not in place, 2) that had ITCs hanging fully extended and/or tied up vs. not in place, and 3) that had washed ITCs correctly or not (see precise definition of these measures in Table 1). Additionally, we looked to see if there were significant differences amongst participants who reported that they would recommend ITCs to friends or family in the future versus those who would not.

A logistic regression model was used to estimate the association between the predictor (independent) variables (socio-demographic variables, as well as variables associated to dengue knowledge, perception of risk, prevention practices, and perceived ITC effectiveness, as described in Table 1) to the dichotomous criterion (dependent) variables of interest associated with correct and consistent use of ITCs. The independent variables for these models included: age, education, occupation, number of children 3 years old and under living at home, wealth index, knows a relative or close friend who had dengue, knows three or more correct symptoms of dengue, knows three or more correct protection methods, knows one or more correct treatment methods, children sleep under a mosquito net, and saw a change in the amount of mosquitoes. The odds ratios and 95% confidence intervals were reported for the adjusted associations estimated through these models.

### Ethics Statement

Our study design received approval from the Institutional Review Boards (IRBs) at the Liverpool School of Tropical Medicine, the Tulane School of Public Health and Tropical Medicine, and the U.S. Naval Medical Research Center Detachment (now Naval Medical Research Unit-6 or NAMRU-6) in Peru. The latter had inter-institutional-IRB agreements with the Tulane School of Public Health and Tropical Medicine and the University of California at Davis. The Regional Health Authority (DIRESA), the local branch of the Ministry of Health, also provided approval.

The trial was registered with the International Standard Randomized Controlled Trial Number Register: ISRCTN08474420. This manuscript does not report the outcome of the cluster randomized trial. The investigation was carried out only on the treatment group; the control group in the study received no treatment. Therefore this study is not an analysis that is dependent on randomized control trial (RCT) design, but instead measures properties of the intervention tool (e.g., ITCs). All subjects provided written informed consent.

The experiments reported herein were conducted in compliance with the Animal Welfare Act and in accordance with the principles set forth in the "Guide for the Care and Use of Laboratory Animals," Institute of Laboratory Animals Resources, National Research Council, National Academy Press, 1996.

## Results

### Participants

A total of 1,742 lots were part of the intervention study area, of which 1,512 (86.8%) were houses. The other lots were vacant houses (188, 10.8%), non-residential (25, 1.4%; e.g., churches, nursery schools, and warehouses), or simply empty lots (17, 1.0%). Of the 1,512 houses, 1,345 (89.0%) wanted ITCs and were considered “study participants;” of the study participants, most (1,333, 99.1%) agreed to participate in the KAP survey. Eighty-two households (4.7%) refused to participate and 85 (4.9%) were not found at their home despite multiple visits. Since this study focuses on correct use of the ITCs, our sample is the intervention homes that completed the KAP survey (593).

The general socio-demographic characteristics of the study population are described in Table 2. Over three quarters of participants were female, and also had less than or equal to 11 years of education. The predominant occupation of respondents was housewife (48.7%). Of the 593 households, 6.2% had one or more pregnant woman living at home, and 20.8% had children 3 years of age or younger living in the home. Overall, households had a median of 5 people (adults and children) living in the home, and a median of 2 children (18 years or under) living in the home.

**Table 2:** Characteristics of KAP respondents in the ITC intervention (n = 593).

Socio-demographic characteristics		Frequency	Percentage
Age		39 (median)	16–81 (range)
Sex			
	Female	455	76.7
	Male	138	23.3
Education*			
	≤ 11 years	467	78.8
	> 11 years	126	21.3
Occupation			
	Housewife	289	48.7
	Merchant/small businessmen	107	18.0
	Unskilled labor	59	10.0
	Health/education professionals	37	6.2
	Manual laborer	35	5.9
	Other occupations**	66	11.1
Household characteristics: other residents			
	Have pregnant woman/women living at home	35	6.2
	Have children ≤ 3 years old living at home	123	20.8
	Number of people living in home	5 (median)	1–16 (range)
	Have children (any age) living at home	2 (median)	0–9 (range)

*In Peru, the sum of years of education in elementary school and high school is equal to 11 years, and post-secondary education may consist of university or technical programs.

**Other occupations include skilled/independent labor, driver, office worker, student, agriculture/ livestock/ fishery, timber merchant, retired, police, and unemployed.

### Hanging, Condition, and Upkeep of ITCs

**Fig 1:**
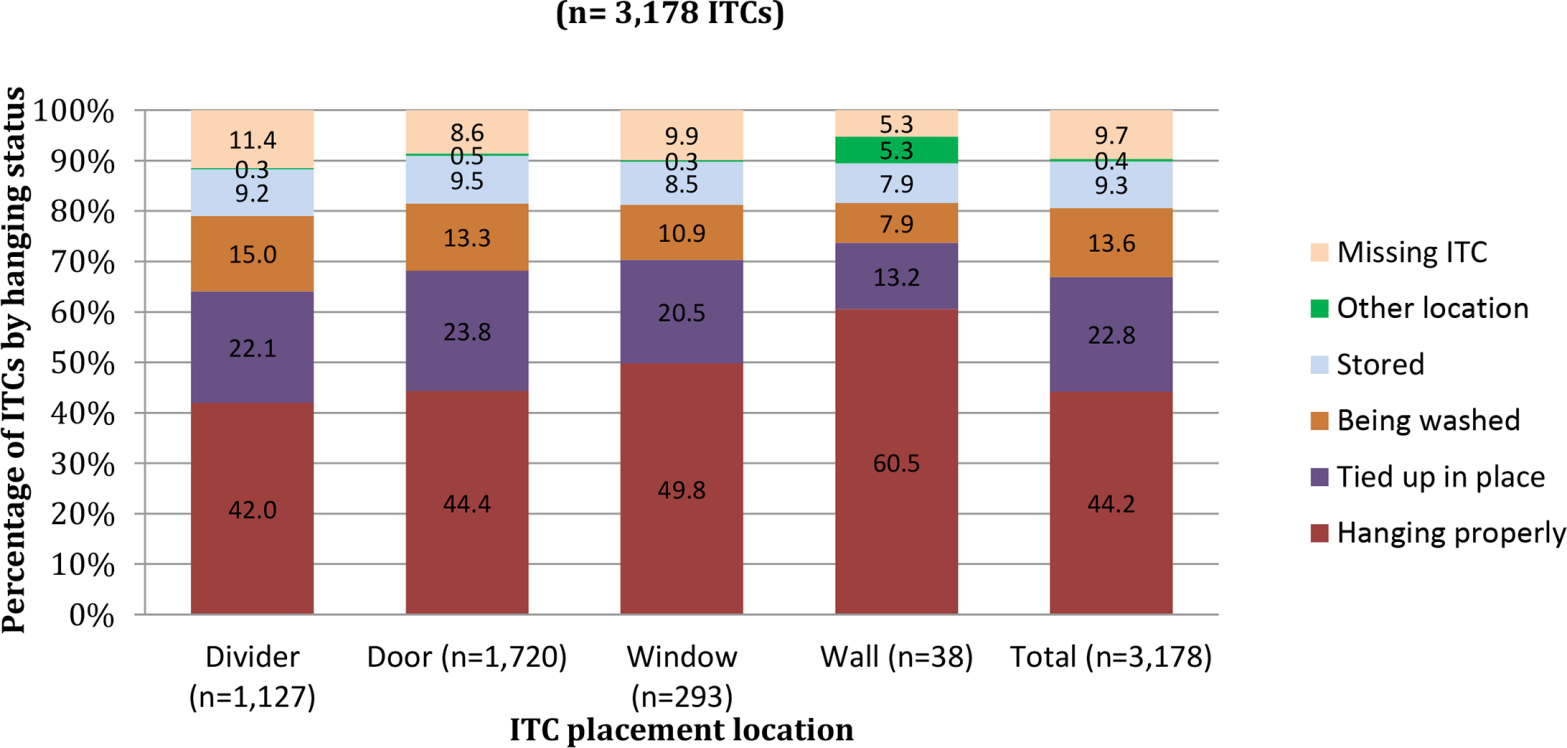
Hanging status of ITCs per location, 9 months after distribution (n = 3,178 ITCs).

**Fig 2:**
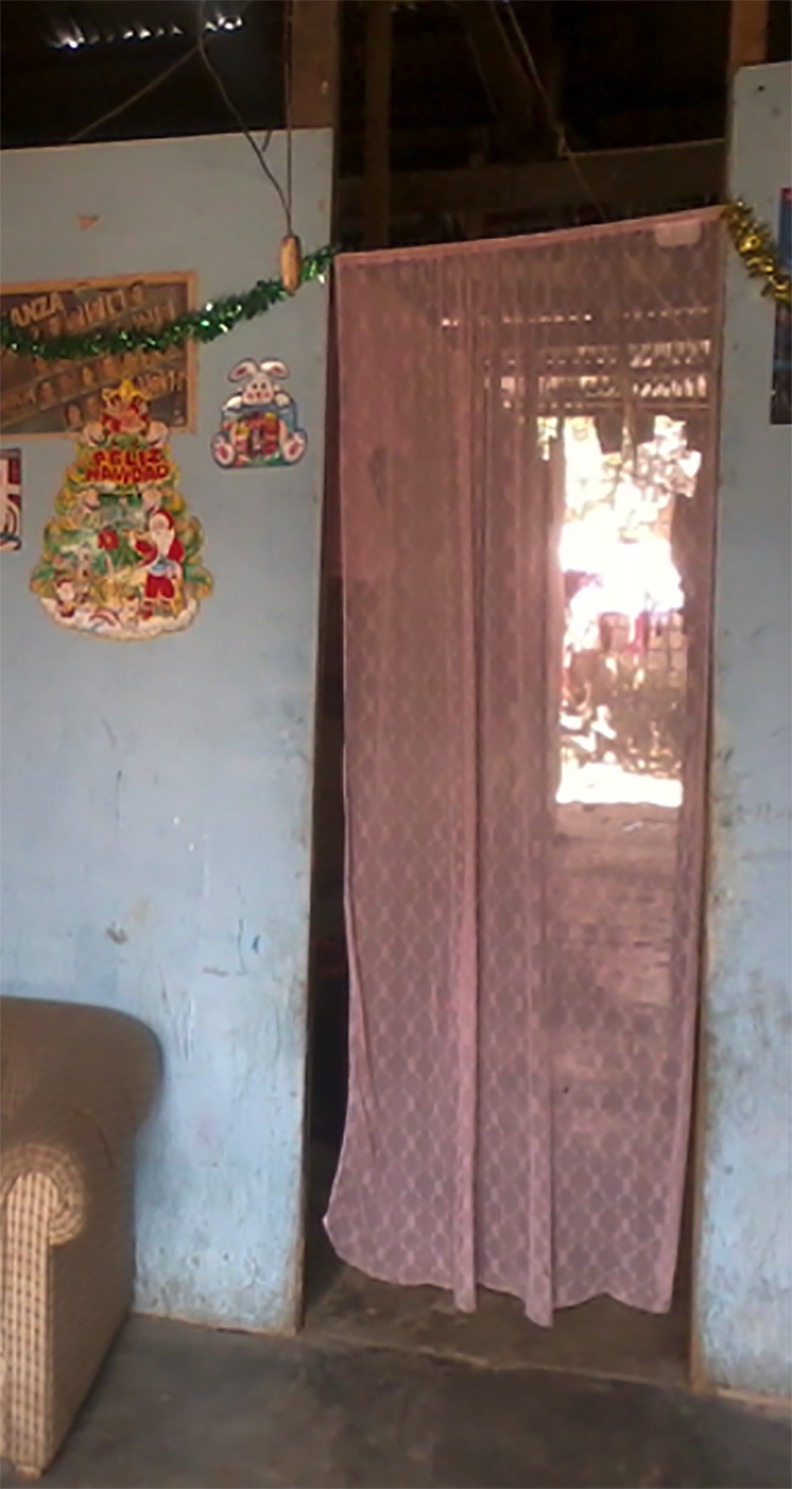
Example of ITC hanging fully extended in doorway.

A total of 3,178 ITCs were distributed to 593 homes in the study area in November and December of 2009. A mean of 5 ITCs (range 1–15 ITCs) were distributed to each home (Table 3). Of these, more than half of the ITCs were hung to cover doorways (n = 1,720) and the remainder were utilized as room dividers (n = 1,127), window coverings (n = 293), or as wall hangings (n = 38) (Fig 1). Examples of ITCs hanging correctly in a doorway can be seen in Fig 2. At 9 months after distribution, approximately 9.7% were missing. Of the 2870 ITCs still present in the home 9 months after distribution, 49% were hanging correctly (i.e. fully extended), 25.2% were hanging in place but tied up, 15.1% were being washed (and our research team had to confirm they saw them in water or hanging to dry), 10.3% were stored somewhere (but observed to be in the home), and 0.5% were being used in a different way than expected (i.e., mosquito net) (Table 3). The fact that 15.1% were being washed indicates that the ITCs were being cared for, although we were unable to verify if the ITCs had been hanging correctly when they were not being washed. A majority of the ITCs, 59.5% (n = 1,707), were reportedly being washed correctly.

**Table 3:** Use, condition and upkeep status of the ITCs 9 months after distribution (3,178 ITCs were distributed to 593 homes, but numbers below are of the 2,870 ITCs that were still in the home at 9 months).

Category	ITC status	% (n)
Distribution	Median number of ITCs distributed per household	5.0
	Range of ITCs distributed per household	1–15
Retention	Percentage of ITCs still in the home at 9 months	90.3 (2,870)
Hanging status	Hanging full extended	49.0 (1,405)
	Tied up	25.2 (724)
	Being washed	15.1 (432)
	Other location	0.5 (14)
	Stored	10.3 (295)
Condition	Excellent state	63.7 (1,829)
	Good state	30.7 (882)
	Poor state	5.5 (159)
Upkeep	Washed correctly	59.5 (1,707)
	Washed incorrectly	23.9 (686)
	Not washed	16.4 (471)
	Mended	0.2 (6)

The average number of hours that ITCs were tied up per day was 10.6 hours with a range of 1–24 hours (18 ITCs were reportedly tied up 24 hours/day). The ITCs placed on walls were most likely to be hanging correctly, compared to those used as room dividers, in doorways, or in windows (Fig 1). A third (n = 1,049) of all ITCs, regardless of original location, were not in place, meaning they were being washed, had been stored, moved to another location, or were missing entirely from the home (Fig 1). All except for 5.5% (n = 159) of the ITCs that were still in use were in either excellent or good condition.

**Fig 3:**
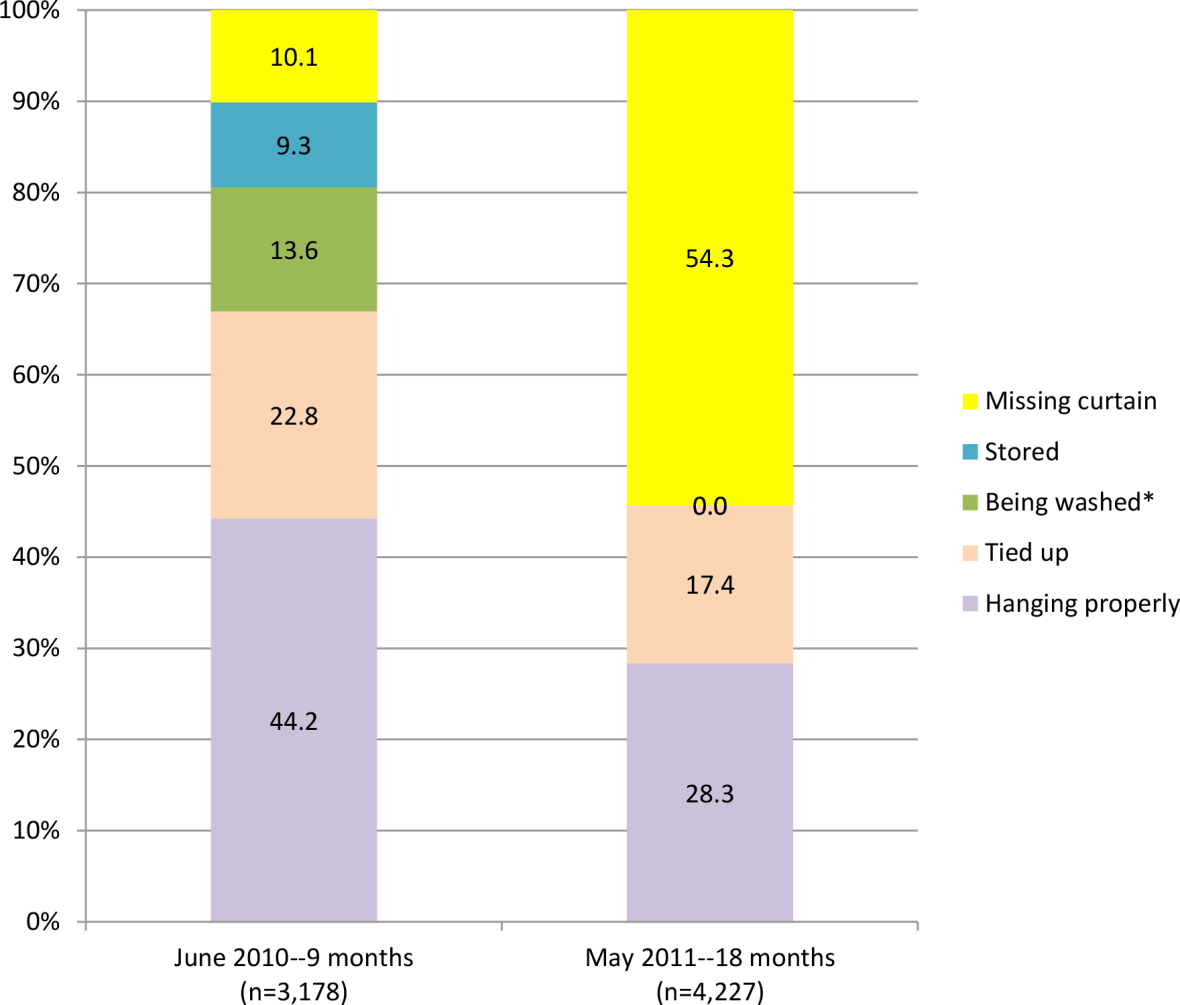
Hanging status of ITCs per location, 9 and 18 months after distribution (n = 3,178 ITCs at 9 months, n = 4,227 at 18 months).

An additional 1,049 ITCs were distributed at 9 months, to replace ITCs that had been lost or damaged (noted as “missing” in the data set, and this included intervention households that took ITCs when they moved or, in some cases, had taken them to another home for protection there), supplement existing ITCs at the request of householders, and offer ITCs to new residents who had recently moved into the study area; this brought the total number of ITCs in the field to 4,227. By 18 months after the original ITC distribution, the percentage of the ITCs hanging correctly or tied up for a portion of the day dropped to 45.8% (n = 1,932) for all locations combined (Fig 3). More than half of the ITCs (51.4%) were missing, and 2.9% of the houses could not be accessed for the final visit.

Perceived effectiveness of the ITCs in our study was high at 9 and 27 months, with 90.2% of respondents at 9 months and 90.6% at 27 months saying they saw a change in the amount of mosquitoes in their home, although for many of these individuals, the change was only seen for a few months (34.9% reported ITCs only worked for a few months at 9 months and 55.7% reported this at 27 months). In addition, the percent that would recommend ITCs to family or friends in the future at 9 months and 27 months was high, at 92.7% at 9 months and 94.6% at 27 months.

### Bivariate Analysis

We examined whether there were significant differences in knowledge, perception of risk, prevention practices, and perceived effectiveness between those households that had ITCs hanging correctly and consistently versus those that did not at 9 months after ITC distribution. A significantly greater (p<0.05) proportion of households that had ITCs hanging properly reported knowing three or more correct symptoms of dengue, knowing someone who had dengue, having children sleep under a mosquito net, and seeing a change in the amount of mosquitoes in the home compared to those that did not have ITCs hanging properly (Table 4). Compared to those who did not, a significantly greater (p<0.05) proportion of households that had ITCs hanging properly and/or tied up for a portion of the day reported knowing at least one correct treatment method for dengue and significantly greater proportion (p<0.01) reported seeing a change in the amount of mosquitoes in the home. Among those households that washed ITCs correctly, a significantly greater (p<0.01) proportion reported being a housewife, knowing three or more correct symptoms of dengue, having children sleep under a mosquito net, and perceiving a change in the amount of mosquitoes compared to those that did not wash correctly. Additionally, a significantly greater (p<0.05) proportion among this same group reported having children ≤3 years at home. Finally, 27 months after distribution, a significantly greater (p<0.01) proportion of those who would recommend ITCs in the future reported having children sleep under a mosquito net and perceiving a change in the amount of mosquitoes in the home compared to those who would not recommend ITCs.

**Table 4:** Factors associated with consistent and recommended future use of ITCs; unadjusted associations based on observation of ITC status at 9 months, using chi-square tests.

		Total	> 50% of ITCs hanging correctly^a^	> 50% of ITCs hanging properly/tied up^a^	> 50% of ITCs washed correctly^a^	Would recommend ITCs ^b^
		% (n = 593)	% (n = 282)	% (n = 428)	% (n = 351)	% (n = 424)
**Socio-demographic**						
	≥36 years old	60.5 (359)	61.4 (173)	61.9 (265)	59.0 (207)	60.3 (240)
	Education (>11 years)	21.3 (126)	21.6 (61)	22.7 (97)	20.2 (71)	20.6 (82)
	Housewife	48.7 (289)	51.1 (144)	49.3 (211)	56.1 (197) **	52.5 (209)
	Pregnant woman living in the home	6.24 (37)	5.3 (15)	6.8 (29)	6.3 (22)	6.0 (24)
	Children ≤3 years at home	20.8 (123)	21.4 (60)	21.8 (93)	24.2 (85) *	23.4 (93)
	Highest (>50%) SES	47.7 (283)	48.2 (136)	48.1 (206)	45.9 (161)	46.7 (186)
**Knowledge of dengue**						
	Knows three or more correct symptoms	77.6 (460)	81.2 (229)*	79.4 (340)	82.3 (289)**	78.6 (313)
	Knows at least one correct treatment	30.5 (181)	33.7 (95)	33.4 (143)*	29.3 (103)	29.7 (118)
	Knows at least 3 correct protection methods	22.4 (133)	20.2 (57)	22.2 (95)	19.9 (70)	20.1 (80)
**Perception of risk**						
	Knows a relative or close friend who had dengue	19.8 (117)	23.6 (66)*	22.1 (94)	21.4 (75)	20.4 (81)
**Mosquito bite prevention practices**						
	Children sleep under a mosquito net	77.6 (460)	81.6 (230)*	78.0 (334)	82.6 (290)**	80.7 (321)**
	Reports doing at least 3 practices to protect against dengue	35.2 (209)	36.5 (103)	35.1 (150)	35.3 (124)	34.9 (139)
**Perceived ITC effectiveness**						
	Perceived a change in amount of mosquitoes in home (at 9 months)^b^	90.2 (526)	92.8 (257)*	92.4 (389)**	93.37 (324)**	-
	Perceived a change in amount of mosquitoes in home (at 27 months)^b^	90.6 (384)	-	-	-	93.5 (372) **

*p<0.05

**p<0.01.

^a^ Reference groups are: for column 2, ≤50% of ITCs not hanging properly (including those observed to be tied up); for column 3, ≤50% of ITCs not hanging in place; for column 4, ≤50% of ITCs not washed correctly (either bleach was used or hung in direct sunlight); and for column 5, would not recommend ITCs.

^b^ “Perceived a change in amount of mosquitoes” and “Would recommend ITCs” were measured at the end of the study (27 months after ITC distribution) compared to the others which were measured at 9 months after ITC distribution. The n for these variables is lower (n = 583 at 9 months and n = 424 at 27 months) due to participants moving out of the study area or not being home at final survey time.

### Multivariate Analyses

Examining the adjusted associations, we found the odds of ITCs hanging correctly (i.e., fully extended) was significantly greater if respondents knew a relative or close friend who has had dengue (OR: 1.66, 95% CI: 1.09, 2.53), or had children that slept under a mosquito net (OR: 1.53, 95% CI: 1.00, 2.33) (Table 5). Those respondents who had their ITCs hung up and/or tied up for a portion of the day also had significantly greater odds of knowing a relative or close friend who had dengue (OR: 1.77, 95% CI: 1.06, 2.96), as well as of knowing at least one correct treatment (OR: 1.65, 95% CI: 1.08, 2.54) and perceiving a change in the amount of mosquitoes in the home (OR: 2.11, 95% CI: 1.19, 3.74). The odds of washing ITCs correctly was significantly greater if respondents were housewives (OR: 1.94, 95% CI: 1.34, 2.81), knew three or more correct symptoms of dengue (OR: 1.94, 95% CI: 1.28, 2.93), had children sleep under a mosquito net (OR: 1.75, 95% OR: 1.15, 2.69), or perceived a change in the amount of mosquitoes in the home (OR: 2.16, 95% CI: 1.22, 3.85). Finally, the odds of recommending ITCs in the future was significantly greater among those respondents who perceived a change in the amount of mosquitoes in the home (OR: 19.78, 95% CI: 7.62, 51.30).

**Table 5:** Factors associated with correct use and recommendation of ITCs: Results from multivariate regression (reporting odds ratio).

	> 50% of ITCs hanging fully extended^a^	> 50% of ITCs hanging/tied up^a^	> 50% of ITCs washed correctly^a^	Would recommend ITCs^c^
	(n = 583)^b^	(n = 583)^b^	(n = 583)^b^	(n = 424)
	OR (95%CI)	OR (95%CI)	OR (95%CI)	OR (95%CI)
≥36 years old	1.22	1.43	1.09	1.04
	(0.86, 1.73)	(0.97, 2.10)	(0.76 1.57)	(0.41, 2.67)
Education (>11 years)	1.12	1.46	1.19	1.07
	(0.72, 1.75)	(0.87, 2.45)	(0.75, 1.88)	(0.32, 3.56)
Housewife	1.18	1.19	1.94 **	1.34
	(0.83, 1.69)	(0.80, 1.77)	(1.34, 2.81)	(0.54, 3.36)
Children ≤3 years at home	1.05	1.36	1.40	1.94
	(0.69, 1.59)	(0.84, 2.20)	(0.89, 2.19)	(0.51, 7.45)
Highest (>50%) SES	0.99	0.96	0.84	0.83
	(0.70, 1.41)	(0.65, 1.42)	(0.92, 1.22)	(0.33, 2.12)
Knows three or more correct symptoms of dengue	1.38	1.35	1.94 **	0.72
	(0.92, 2.07)	(0.87, 2.09)	(1.28, 2.93)	(0.24, 2.17)
Knows at least one correct treatment	1.26	1.65 *	0.88	0.80
	(0.88, 1.81)	(1.08, 2.54)	(0.60, 1.28)	(0.31, 2.06)
Knows a relative or close friend who has had dengue	1.66 **	1.77 *	1.45	1.24
	(1.09, 2.53)	(1.06, 2.96)	(0.92, 2.27)	(0.37, 4.11)
Children sleep under a mosquito net	1.53 *	1.18	1.75 **	3.45
	(1.00, 2.33)	(0.74, 1.87)	(1.15, 2.69)	(1.28, 9.26)
Perceived a change in the amount of mosquitoes in the home at 9 months	1.69	2.11 *	2.16 **	19.78 **
	(0.95, 3.02)	(1.19, 3.74)	(1.22, 3.85)	(7.62, 51.30)

*p<0.05

**p<0.01.

^a^ Reference groups are, for column 2, ≤50% of ITCs not hanging properly (including those that were observed to be tied up); for column 3, ≤50% of ITCs not hanging in place; for column 4, ≤50% of ITCs not washed correctly (either bleach was used or hung in direct sunlight); and for column 5, would not recommend ITCs.

^b^ The total number of participants in this analysis (n = 583) is slightly lower than those who participated in the KAP baseline (n = 593) because one variable in the multivariate logistic regression was obtained at 9 months, and 10 individuals were not available to respond.

^c^ This variable was measured at the end of the study (27 months after ITC distribution) compared to the others which were measured at 9 months after ITC distribution. The n in this column is lower due to participants moving out of the study area or not being home at final survey time.

## Discussion

Our analysis revealed that perceived effectiveness (e.g. seeing a change in the amount of mosquitoes in the home) was significantly associated with two of our measures for correct and consistent use: having the ITCs hanging (even if tied up) and washing the ITCs correctly (Table 5). However, despite the ease of use of the ITCs, the use of the ITCs decreased from 68.6% at 9 months to 45.7% at 18 months after distribution. Two similar studies–one in Venezuela and Thailand on ITCs for dengue prevention, and another in Ethiopia on ITNs for malaria prevention–also found a decrease in usage over time, and found that long-term use of the ITCs was associated with perceived effectiveness: when participants perceived that their ITNs had lost effectiveness, they stopped using them [21,28]. Although the perception of effectiveness was high in our study at 9 and 27 months (over 90% reporting they felt ITCs worked well at both times), there was a high percentage of participants who perceived that the change in the amount of mosquitoes lasted only a few months (34.9% at 9 months, 55.7% at 27 months), which might have affected use or explain why so many ITCs were no longer in place at 18 months. However, the association between perception of effectiveness and future use of the ITCs was still significant at 27 months after distribution, and the odds of recommending ITCs to friends or family in the future was significantly higher among those who did perceive the ITCs to be effective (Table 5).

Controlling for various socio-demographic and knowledge-related variables, knowing a relative or close friend who had dengue previously and using a mosquito net were both positively associated with our most important measure of correct and consistent ITC use: having 50% or more of the ITCs in that house hanging fully extended or tied up. Previous research also found that the correct and consistent use of ITCs was positively associated with knowing someone who had been infected with dengue [15]. It is possible that perceiving that dengue could happen to anyone–including themselves—or seeing firsthand the severity of illness that can result from a dengue infection served as an incentive to use the ITCs correctly [41]. In addition, we also found that in the adjusted models, knowing someone who has had dengue and knowledge of dengue symptoms were both associated with the odds of washing the ITCs correctly–one of our measures of correct use. Previous studies on the acceptability and use of ITNs as a vector control tool for malaria have not shown an association between knowledge of the infection and use of the control measure [19,21,22]. Also, a previous study of ITCs in Thailand and Venezuela did not find a significant association between knowledge of dengue and ITC use [28]. However, in the other studies, knowledge of dengue (or malaria) was examined in association to use of the ITCs or ITNs, and like those studies, we did not see an association with ITC correct use either–we only saw the association between knowledge of dengue symptoms and washing the ITCs correctly, a measure not used in those studies.

This study also presents novel KAP and socio-demographic factors positively associated with the correct and consistent use of ITCs: having children sleep under a mosquito net and being a housewife. We suspect that participants whose children slept under mosquito nets at night were more familiar with using vector-control tools, and that these are families that tend to practice preventive behaviors, and for these reasons, were more likely to use ITCs correctly and consistently—selection bias. Moreover, the choice of a mosquito net indicated familiarity and acceptability of an insecticide treated material for vector control–similar to an ITC for dengue control. In future health campaigns associated to ITCs, these families would likely be “early adopters” of an ITC intervention, and families who would likely require less health promotion to initiate ITC use. The positive association between washing ITCs correctly and being a housewife could be due to the fact that those who did the washing for the home were predominantly housewives and were therefore more likely to be familiar with washing items correctly.

### Limitations

There were several limitations to this study. First, we were unable to track any changes in the location of individual ITCs inside the home. For example, were ITCs placed in the doorway more likely to be moved to other locations compared to those placed on the wall? These data would have enabled us to determine the most successful long-term placement for ITCs within the home. Second, it would have been useful to know more about maintenance behaviors: how often did individuals report washing the ITCs? How often did these need to get mended? Though we perceive the ITCs to require minimal upkeep, it would be useful to find out what type of upkeep effort was required. Third, it would have been interesting to quantify how much of an impact was attributable to a barrier effect and how much was attributable to an insecticide effect. However, the trial did not aim to measure the impact of insecticide vs. non-insecticide treated curtains; rather, the aim was to assess the impact of ITCs as a single intervention vs. the status quo which was no ITCs at all. Fourth, “correct and consistent use” was a subjective measure used for this study and based on only two observation visits. It is possible that ITCs that were coded as “hanging loosely and extended” were actually tied up at other times of the day, and vice versa. Despite this limitation, we felt it was important to assess use in the most objective way we could: observation of use (vs. self-reporting). We also expect that if ITCs were hanging—fully extended or not—that they were hanging there most of the time, since participants did not know when we were coming to monitor the use of ITCs and we would not expect them to put them in place just for us. Finally, due to social desirability bias (wanting to seem like they were using them correctly most of the time), respondents might be more likely to underestimate the time the ITCs were tied up.

### Conclusion

ITCs are an attractive vector control intervention due to the minimal upkeep and attention they require. The findings reported here add to the body of literature regarding the effectiveness of ITCs as a vector control tool in dengue endemic areas. The long-term use of ITCs in this study decreased significantly over time; we believe this was primarily the result of a reduction in perceived effectiveness of the insecticide. In fact, this perception was correct: when we learned that people were perceiving the ITCs to no longer be working, we removed several ITCs from the field, tested them using standard WHO cone bioassays [42], and found that indeed, the ITCs were not killing mosquitoes as they should have been. As such, we then decided to remove all ITCs from the field to re-impregnate them, which might have validated people’s perceptions that the ITCs lost their effectiveness. The odds of using ITCs correctly and consistently and recommending ITCs in the future were significantly greater among those who did perceive the ITCs to be effective. Hence, importantly, despite the fact that the effectiveness of the ITCs did decline noticeably, it is encouraging that 18 months after distribution, ITCs were still hanging in almost half of the houses.

It is important for future ITC intervention programs to note that, although all participants had a choice as to how many and where to place their ITCs, a higher percentage of ITCs that were hung on the wall were hanging properly at 9 months as compared to windows, doors, or room dividers (Fig 3). This study supports previous findings that perception of ITC effectiveness was associated with correct and consistent use. Additionally, this study reports that certain participant characteristics and behaviors—especially knowing someone who has had dengue or having children sleep under a mosquito net—were significantly positively associated with different measures of correct and consistent use of ITCs. These findings reveal that perception of risk for dengue (by knowing someone who has had dengue) and already practicing preventive behaviors are key factors associated in using ITCs correctly and consistently, and that vector control health promotion should include messages stressing the high risk for all to dengue in dengue endemic regions like Iquitos. Finally, findings from this study could guide future ITC-related interventions regarding locations in the house where ITCs were more likely to be used correctly and remain over time.

## Supporting Information

S1 Checklist STROBE checklist. (DOC)
