# Correction: Factors Associated with Correct and Consistent Insecticide Treated Curtain Use in Iquitos, Peru

**DOI:** 10.1371/journal.pntd.0004647

**Published:** 2016-04-19

**Authors:** Valerie A. Paz-Soldan, Karin Bauer, Amy C. Morrison, Jhonny J. Cordova Lopez, Kiyohiko Izumi, Thomas W. Scott, John P. Elder, Neal Alexander, Eric S. Halsey, Philip J. McCall, Audrey Lenhart

The affiliation for the tenth author is incorrect. Philip J. McCall is affiliated with the Liverpool School of Tropical Medicine, Liverpool, United Kingdom.
