# Supplementary material for: Factors Associated with Correct and Consistent Insecticide Treated Curtain Use in Iquitos, Peru
Source: PLoS Negl Trop Dis. 2016 Mar 11;10(3):e0004409. doi: 10.1371/journal.pntd.0004409 (PMC4788147; doi:10.1371/journal.pntd.0004409)
Supplement: S1 Checklist — (DOC) [file pntd.0004409.s001.doc]

S1 Appendix. STROBE Checklist

|  | Item No | Recommendation |
| --- | --- | --- |
| **Title and abstract** | 1 | Indicate the study’s design with a commonly used term in the title or the abstract  **In the abstract, the study was described as “observational”.** |
|  |  | (*b*) Provide in the abstract an informative and balanced summary of what was done and what was found  **The abstract contains an informative summary of the design of the study (i.e., hanging ITCs in 593 houses, nothing in controls); KAP baseline survey weeks before ITCs were distributed; and ITC monitoring checklist at 9, 18 and 27 months. The main results are reported.** |
| Introduction | | |
| Background/rationale | 2 | Explain the scientific background and rationale for the investigation being reported  **Done.** |
| Objectives | 3 | State specific objectives, including any prespecified hypotheses  **Done in the last paragraph of the introduction: the objective of this study was to determine the individual and household-level socio-demographic factors that are associated with the correct and consistent use of ITCs in Iquitos, Peru. Correct and consistent use was defined for this study as 1) ITCs *observed* as hanging properly—loosely and extended—at the time of visit, 2) ITCs *observed* to be tied up, but still in their place, at the time of visit, and 3) ITCs *reported* as washed correctly – with only water and/or mild soap and hung to dry in the shade. This was an observational study with no pre-specified hypotheses.** |
| Methods | | |
| Study design | 4 | Present key elements of study design early in the paper  **Done under methods section – starting in second paragraph.** |
| Setting | 5 | Describe the setting, locations, and relevant dates, including periods of recruitment, exposure, follow-up, and data collection  **Setting described in the first paragraph of the methods section. Dates of surveys and ITC distribution and follow up data collection described in the study design section.** |
| Participants | 6 | (*a*) *Cohort study*—Give the eligibility criteria, and the sources and methods of selection of participants. Describe methods of follow-up  *Case-control study*—Give the eligibility criteria, and the sources and methods of case ascertainment and control selection. Give the rationale for the choice of cases and controls  *Cross-sectional study*—Give the eligibility criteria, and the sources and methods of selection of participants  **Described in study design: as part of a larger cluster-randomized controlled trial, there were 10 treatment and 10 control clusters of approximately 70 households each. All participants in the treatment clusters could request as many ITCs as they wanted. The data for this analysis come from the 593 households in the treatment cluster.** |
|  |  | (*b*) *Cohort study*—For matched studies, give matching criteria and number of exposed and unexposed  *Case-control study*—For matched studies, give matching criteria and the number of controls per case  **Not applicable.** |
| Variables | 7 | Clearly define all outcomes, exposures, predictors, potential confounders, and effect modifiers. Give diagnostic criteria, if applicable  **Variables of interest are described in text (methods section, under subsection “monitoring of ITC use”), as well as in Table 1.** |
| Data sources/ measurement | 8* | For each variable of interest, give sources of data and details of methods of assessment (measurement). Describe comparability of assessment methods if there is more than one group  **Done in study design and “monitoring of ITC use” sections.** |
| Bias | 9 | Describe any efforts to address potential sources of bias  **Described in last sentence of limitations in discussion section.** |
| Study size | 10 | Explain how the study size was arrived at  **As described in the first paragraph of the study design section, “This study is part of a larger cluster-randomized controlled trial, initiated in October 2009, to measure whether ITCs can reduce dengue transmission and dengue vector activity in 10 treatment clusters compared to 10 control clusters of approximately 70 households each (2-3 city blocks).” This study was an observational study of those who received the ITCs: 593 households.** |
| Quantitative variables | 11 | Explain how quantitative variables were handled in the analyses. If applicable, describe which groupings were chosen and why  **Described in “Data Analysis” section of study design, as well as “Monitoring of ITC Use”. Also some discussion about the groupings or alternative measures that could be used in future research discussed in the discussion section.** |
| Statistical methods | 12 | Describe all statistical methods, including those used to control for confounding  **Done in “Data Analysis” section.** |
|  |  | (*b*) Describe any methods used to examine subgroups and interactions  **Not applicable** |
|  |  | (*c*) Explain how missing data were addressed  **Not applicable** |
|  |  | (*d*) *Cohort study*—If applicable, explain how loss to follow-up was addressed  *Case-control study*—If applicable, explain how matching of cases and controls was addressed  *Cross-sectional study*—If applicable, describe analytical methods taking account of sampling strategy  **Not applicable** |
|  |  | (*e*) Describe any sensitivity analyses |

Continued on next page

| Results | | |
| --- | --- | --- |
| Participants | 13* | Report numbers of individuals at each stage of study—eg numbers potentially eligible, examined for eligibility, confirmed eligible, included in the study, completing follow-up, and analysed  **See first paragraph under “participants” under “results”. Moreover, details are included throughout the manuscript to make this clearer.** |
|  |  | (b) Give reasons for non-participation at each stage  **Described in the paragraph above.** |
|  |  | (c) Consider use of a flow diagram  **Decided not to because we think text is clear enough.** |
| Descriptive data | 14* | Give characteristics of study participants (eg demographic, clinical, social) and information on exposures and potential confounders.  **Done in first paragraph of results section and in Table 2.** |
|  |  | (b) Indicate number of participants with missing data for each variable of interest  **In most tables we report data based on the households (tables 2, 4 and 5), where the n=593 (houses in treatment cluster). For table 3, we report the conditions of each ITC in the houses that were available. 3178 ITCs were distributed, but 2870 ITCs were in people’s homes 9 months later, as stated in the heading.** |
|  |  | (c) *Cohort study*—Summarise follow-up time (eg, average and total amount)  **NA** |
| Outcome data | 15* | *Cohort study*—Report numbers of outcome events or summary measures over time |
|  |  | *Case-control study—*Report numbers in each exposure category, or summary measures of exposure |
|  |  | *Cross-sectional study—*Report numbers of outcome events or summary measures  **This is done in the results section, Table 3, as well as Figures 1 and 3.** |
| Main results | 16 | Give unadjusted estimates and, if applicable, confounder-adjusted estimates and their precision (eg, 95% confidence interval). Make clear which confounders were adjusted for and why they were included  **Table 4 presents unadjusted estimates and Table 5 reports adjusted estimates with 95% CI – and most relevant results highlighted in text of results section.** |
|  |  | (*b*) Report category boundaries when continuous variables were categorized  **Not applicable** |
|  |  | (*c*) If relevant, consider translating estimates of relative risk into absolute risk for a meaningful time period  **Not applicable** |
| Other analyses | 17 | Report other analyses done—eg analyses of subgroups and interactions, and sensitivity analyses  **Not applicable** |
| Discussion | | |
| Key results | 18 | Summarise key results with reference to study objectives  **The first sentence of the discussion repeats the main finding, and the following paragraphs discuss other key variables associated to correct ITC use.** |
| Limitations | 19 | Discuss limitations of the study, taking into account sources of potential bias or imprecision. Discuss both direction and magnitude of any potential bias  **Limitations discussed in discussion section.** |
| Interpretation | 20 | Give a cautious overall interpretation of results considering objectives, limitations, multiplicity of analyses, results from similar studies, and other relevant evidence  **This is done in the context of other literature as well.** |
| Generalisability | 21 | Discuss the generalisability (external validity) of the study results  **Our discussion presents our findings within the context of other similar studies, contributing to the literature on this subject, and suggesting other measures to be considered in future studies.** |
| Other information | | |
| Funding | 22 | Give the source of funding and the role of the funders for the present study and, if applicable, for the original study on which the present article is based  **This is done.** |

*Give information separately for cases and controls in case-control studies and, if applicable, for exposed and unexposed groups in cohort and cross-sectional studies.

**Note:** An Explanation and Elaboration article discusses each checklist item and gives methodological background and published examples of transparent reporting. The STROBE checklist is best used in conjunction with this article (freely available on the Web sites of PLoS Medicine at http://www.plosmedicine.org/, Annals of Internal Medicine at http://www.annals.org/, and Epidemiology at http://www.epidem.com/). Information on the STROBE Initiative is available at www.strobe-statement.org.
